# Clinical Thermometry

**Published:** 1875-05-15

**Authors:** B. Kraus


### CLINICAL THERMOMETRY.

FROM THE "COMPENDIUM DER NEUEREN MEDICINISCHEN WISSENSCHAFTEN," BY DR. B. KRAUS.

*Condensed and translated by Dr. H. Gradle.*

### PRECAUTIONS TO BE OBSERVED IN THE APPLICATION OF THE THERMOMETER.

ON resorting to the axilla as place of application, the gathered perspiration must first be removed, and the skin dried. If now the axilla be firmly closed, the time in which the mercury reaches its highest point, is, as Liebermeister has shown, reduced to three or four minutes. The supine position is not indispensable, as long as the patient is in a position permitting of proper application and inspection of the thermometer, though it is often preferable. The instrument is now slightly warmed by grasping with the hand, hereupon inserted, and the axilla closed by pressing the arm against the chest; if the patient is restless or somnolent, or even much emaciated, it may be necessary to support the instrument with the hand, or at least to observe that it is kept in place. The mercury reaches its acme rarely under ten minutes, sometimes not before twenty minutes. If precision is desired, it may be necessary to allow the thermometer to remain in place for a few minutes, even after the maximum is attained. Duclos considers the temperature indicated by the thermometer as correct, if it remains stationary in a uniform external temperature; a rather hazardous statement, as the animal temperature may change steadily. Alvarenga keeps the instrument in place for fifteen minutes, and registers the temperature three minutes after the mercury ceases to rise. ("Grundzuege der allg. klin. Thermom. etc.," von Dr. P. F. Alvarenga. Aus dem *Portug. Uebers.*, von Dr. O. Wucherer, 1873.)

On measuring the temperature in the vagina or rectum, the well constructed, *oiled* thermometer ought to be introduced to the distance of about six centimetres. Thomas advises to heat the instrument previously to a temperature 1° to 2° above the point to be expected, enabling one to obtain a practically correct result in one-fourth to one-half minute.*

It is, of course, necessary to note all previous accidents which may have influenced the temperature, as violent pain, vomiting, defecation, hæmorrhage, perspiration, etc. Nor can we neglect to take record of the time of the day at every observation; an observation, however, of the external temperature, and atmospheric tension is not necessary for practical purposes. In summary, our data are only reliable if the thermometer adapts itself well to the parts explored, all cooling influences are avoided, the instrument is kept in place for twenty minutes, and the degree only noted sometime after the maximum has been attained.

* Of course, not applicable with self-registering instruments.

NUMBER AND TIME OF THERMOMETRIC OBSERVATIONS.

Under ordinary circumstances, it is advisable to register the temperature of different days at nearly the same hour, repeating this twice daily, preferably between 7 and 9 A.M., the time of the maximum, and 4 and 6 P.M., the period of minimum temperature. But if exacerbations and remissions occur at another time, the observations ought to take place accordingly.

In important cases, or when a special object is sought, the temperature must be registered every two to four hours, as in severe acute diseases, especially at first, in order to ascertain the time of exacerbations, besides in cases of doubtful diagnosis, or when deviations from the usual course occurs; in short, whenever anything strange is observed. If it is merely desired to follow accurately the course of the temperature in any disease, four to six observations should be made; for instance, at 7 to 8, 9 to 10 A.M.; 12 M.; 3 to 4, 6 to 7, 10 to 11 P.M.; and whenever any irregularity occurs, another observation may be made early in the morning.

When sudden changes of temperature occur in a disease, for instance, at a rapid crisis, or in intermittent fever, hourly, half-hourly, or still better, continuous record will illustrate the changes. This, however, cannot be demanded in private practice, but is necessary when the laws of a disease are to be determined.

GRAPHIC RECORD OF TEMPERATURE.

For the utilization of any sort of thermometric observations, it is indispensable to note the continuous record; but in order to take in the course of the temperature at one glance, the graphic method is by far the best; in other words, the construction of tables giving the thermometric course in form of a curve.

In order to facilitate the reading, the table may be divided into the degrees of both the centigrade and Fahrenheit scale; the tables may be rendered more complete by the construction, also, of curves, of the frequency of the respiration and pulse, in different inks. If now important accidents, therapeutic measures, etc. are noted at their proper place, a complete history of the disease is thus obtained. (Here follows a description of the graphic tables of Duclos, Wunderlich and Alvarenga, with illustrations, for which we must refer to the original.)

PHYSIOLOGICAL TEMPERATURE AND ITS OSCILLATIONS.

The oscillations of temperature in the normal state are altogether minimal, and whether spontaneous or caused by extraneous influences, are always transitory. Whenever a deviation occurs in either direction, the corresponding reaction is not slow to appear. According to Wunderlich, the normal oscillations rarely exceed one-half degree C. in any one individual; in fact, this *stability of temperature is ordinarily a sign of health.* The same authority considers a temperature above 37.5° C. (99.5° F.) or below 36.25° C. (97.25° F.) as suspicious. Crampton probably was incorrect when he stated that a daily temperature of 99° F. or more indicated disease. Possibly, this may hold good for London. At any rate, Alvarenga found a good many individuals in Lisbon, who fared well at that temperature, or if subject to disease, the latter was of a chronic nature. Whenever the production of animal caloric is increased in the normal body, a corresponding augmentation of the loss does also occur, and besides the production itself is diminished for some time; if, however, the latter is depressed too much, the losses are correspondingly reduced; and again, if these are too low, the production of heat decreases likewise, while, if the opposite occurs, an augmentation of production is the result. As long as the body is in a state of health, every disturbance of this nature remedies itself.

The variations of temperature are, according to Alvarenga, either general or local, the mean normal temperature being 37° C. (98.6° F.) But while investigators do not all agree as to this number, the difference is extremely slight. Thus Davy states 37.33° under the tongue, 37° C. in the axilla; Despret, 37.9°; Roger, 37.2° in children; Wurtz, 37°, and in the axilla, 36.54°; Baerensprung, 37.8°; Billroth, 37.5° as average, and the limits from 36.3° to 37.9° in two hundred observations; Chisholm, 36.11° (average of sixty-seven individuals); Froehlich and Lichtenfels, 36.9°; Mignon, 37°; Robert Latour, 36.5° to 37.5° (average 37°); Maurice, 36.20°; Hardy, 36.5° to 37.5°, and in early childhood, 37.1°; Compton, 97.4° F., limits 95.5° to 98.5°; Hirtz, 36° to 38°; Anfrun, 37° to 37.5°; Wunderlich 37°, limits 36.25° to 37.5°; Jaccoud, 37.2° to 37.5°; Alvarenga, 37.27°.

Henry Roger found the following average in nine children: At birth, 36.14°; first to seventh day, 37.08°; four months to six years, 37.11°; six to fourteen years, 37.31°; which was an average of 36.91° from birth to the fourteenth year, and from 37.2° to 37.17° from the first day until that period.

The temperature of internal organs is also yet a matter of dispute; Davy, Breschet and Bequerel consider the temperature increasing as the circulatory centre is approached; the warmest place being the left ventricle (Davy); but according to Cl. Bernard, the blood of the left ventricle is colder than that of the right side of the heart.* The skin is the coldest part of the body, on account of the constant loss by radiation and evaporation. According to P. Beer, its temperature varies between 32° and 37° C. Martin found the cutaneous temperature over the abdomen, 28° to 30° R. (95° to 98.5° F.); over the chest, 26.4° to 27.1° (91.4° to 93.2° F.); in the hand, 23.2° to 29.6° (84.7° to 98.6° F.); and at the foot, 16° to 27° (68° to 93° F.) Marey, Donders, and others claim that the internal temperature rises when the external temperature sinks, and *vice versa.*

The normal temperature may vary, though within narrow limits, according to different circumstances.

The influence of age can be seen from the following table by Baerensprung:

At birth, 37.08°; after birth, 36.95°; within the first ten days, 37.55°; until puberty, 37.63°; from 15 to 20 years, 37.39°; from 21 to 30 years, 37.08°; from 31 to 40 years, 37.11°; from 41 to 50 years, 36.94°; from 61 to 70 years, 37.09°; from 80 years, 37.46°.

The time of the day also is of some influence; during the day time, an increase; during the night, a decrease occurs. From a diurnal table by Baerensprung, we abstract the following:

* Bernard's researches are entitled to most consideration.

The highest temperature of adults is observed in the afternoon, a few hours after eating, from 4 to 8 P.M.; the lowest after midnight, from 2 to 4 A.M. (36.31°); from 12 to 2 P.M. the temperature ranges at 36.65°; from 5 to 7 A.M. at 36.69°; the average from 12 P.M. to 7 A.M. being 36.55° C. The first maximum temperature occurs in the morning from 9 to 11 A.M. (37.26°); the second in the afternoon from 4 to 6 P.M. (37.26°); the first minimum is noticed at 5 to 7 A.M. (36.60°); the second, 8 to 10 P.M. (37.03°). From 7 A.M., the temperature rises until midnight, with the exception of a slight reduction before dinner, to sink again until 5 A.M., when a very slight increase is observed until breakfast.

Besides age and time of day, an influence is also claimed for the external temperature, the climate, and the seasons, but its exact value is not yet determined. Some claim that the temperature is about one degree higher in tropic countries; others (Edward) found no difference, not even any between summer and winter. An external temperature of 212° F., was not observed by Blagden to be of any influence on the animal heat. Another factor is muscular activity; but Baerensprung found that *cæteris paribus* sleep did not alter the human temperature, which Alvarenga explains by the absence during sleep of circumstances abstracting heat. A reduction in the quantity or quality of food results in a reduction of temperature, but only after the lapse of a few days. Sex, constitution and temperament, are not known to influence animal temperature in any manner.

[*To be continued.*]

